# ‘Relearning how to think’: A brief online intervention to modify biased interpretations in emotional disorders—study protocol for a randomised controlled trial

**DOI:** 10.1186/s13063-021-05459-3

**Published:** 2021-07-31

**Authors:** Inés Nieto, Carmelo Vazquez

**Affiliations:** Department of Clinical Psychology, School of Psychology, Complutense University of Madrid, 28223 Madrid, Spain

**Keywords:** Cognitive Bias Modification, Ambiguous interpretations, Emotional disorders

## Abstract

**Background:**

Cognitive biases play an important role in the development and maintenance of emotional disorders, such as depression and anxiety. Novel procedures, known as Cognitive Bias Modification (CBM), aim to reduce these dysfunctional information processing modes. This study develops a brief clinically based online intervention programme to modify biased interpretations in depression and anxiety (CBM-I_Clin_), overcoming some methodological issues that have been addressed in previous literature.

**Methods:**

Volunteer participants will be recruited via social media and posters at the university. They will be randomly assigned to an experimental group or a waiting list control group. Both groups will complete two assessment sessions (before and after the intervention) consisting of questionnaires measuring cognitive and emotional variables as well as experimental tasks measuring cognitive biases (i.e. attention, memory, and interpretation). After the first assessment session, only participants in the experimental group will receive a link to follow the four CBM-I_Clin_ sessions at home. All participants will receive, via email, follow-up questionnaires 2 weeks and 3 months after the second assessment.

**Discussion:**

This study will test the 'Relearning how to think', an online programme potentially beneficial to modify cognitive biases in emotional disorders. Several limitations of previous CBM procedures are addressed, and the impact of the programme both on objective cognitive bias tasks and clinical symptoms will be explored.

**Trial registration:**

ClinicalTrials.gov NCT03987477. Prospectively registered on June 17, 2019

## Background

Anxiety and mood disorders are the most common mental disorders in the general population, showing an 18.1% and 9.5% 12-month prevalence, respectively [1]. Both disorders are associated with high social and economic costs, as well as high rates of chronicity and relapse [2, 3].

High patterns of comorbidity between anxiety and mood disorders are consistent across studies [1, 4]. This comorbidity could be due, at least in part, to the fact that these disorders share transdiagnostic factors such as rumination [5] and are also likely to share some general risk factors [6]. Cognitive theories point to dysfunctional thinking as one of the main variables related to the onset and maintenance of these emotional disorders [6–8] According to these influential theories, depressed and anxious individuals show specific cognitive dysfunctions that can ultimately lead to symptom development [9, 10]. Several types of dysfunctions have been distinguished in regard to the onset and maintenance of emotional disorders. A useful heuristic to clarify diverse types of cognitive activity intervening in psychopathology is the so-called cognitive taxonomy, initially proposed by Ingram and Kendall [11]. This taxonomy differentiates between *structural* variables (i.e. broad cognitive schemas through which information is filtered, represented, and organised), *operational* variables (i.e. the mechanisms such as attentional, interpretation, or memory biases, by which cognitive structures work), and *product* variables (i.e. the tangible outputs such as thoughts, images, and memories, with which clinicians typically work with their clients). In general, cognitive biases have been defined as errors or distortions related to the operational domain that occur systematically at different times and across distinctive situations, reflecting ‘irrational’ modes of perceiving and elaborating information [12, 13].

### Cognitive Bias Modification

*Cognitive Bias Modification* (*CBM*) is a recent approach developed to alter cognitive biases and explore the link between them and symptom development. CBM procedures have mainly been aimed at modifying attentional, memory, or interpretation biases. Besides their clinical utility, these procedures are theoretically ambitious as their rationale is that a causal link between cognition and emotion would be demonstrated if changes in cognition are accompanied by consequent changes in emotion. Thus, the efficacy of CBM would provide support to the etiological role of cognition in emotional disorders. Although it has been applied in different conditions, such as eating disorders, substance abuse, or anger-related problems [14], the main focus has been on anxiety and depression [15–18].

Mathews and Mackintosh [19], in their pioneer study in the field of CBM for interpretation processes (CBM-I), used a paradigm to induce negative interpretation biases in healthy individuals and found a possible causal link to anxiety. Since then, studies using different training paradigms have found an association between change in interpretation bias and symptoms. Recent meta-analyses show evidence of a correlation between change in interpretation bias and negative mood [20], and significant differences between CBM training and control groups in anxiety and depression measures [17]. Some transdiagnostic factors, such as rumination, have also been found to improve with CBM-I [21]. A review of 12 meta-analyses of CBM procedures [18] revealed that there was a significant effect in 8 out of 10 meta-analyses for anxiety and 3 out of 7 meta-analyses for depression. Authors also found significant changes in attention biases in 8 out of 9 meta-analyses of studies using Attentional Bias Modification procedures (ABM) and changes in interpretation biases in the 3 meta-analyses on CBM-I. All this evidence reflects a possible causal link between cognitive biases and symptoms that, at least in the case of interpretation biases, might suggest the existence of a causal connection with a depressed mood in particular [22].

Although results are promising, there are some limitations in the current CBM procedures [23, 24]. First of all, some variables could affect the efficacy of CBM but remain unexplored. For example, although some studies are indicating that the use of mental imagery during CBM procedures could have a beneficial impact on CBM [25–27], it is still unclear whether this factor is relevant [17, 20]. Also, most CBM procedures are based on the idea that repetitive exposure to a specific way of processing information leads to its automatic use later in daily life [28]. Yet, the theoretical support to this mechanistic and repetitive procedure (that typically involves hundreds of trials) is still not clear. In fact, it could be possible that CBM procedures focused on enhancing elaborative rather than automatic processing modes could be more beneficial for disorders like depression where those elaborative mechanisms seem to be more affected than automatic ones [26].

Another methodological recommendation to improve CBM paradigms is the use of direct and indirect measures to evaluate cognitive change [14, 22]. This involves asking participants to directly respond to a series of interpretations (e.g. plausibility ranking or the scrambled sentence task) together with non-conscious measures of interpretations (e.g. reaction time). The evaluation of long-term benefits, when participants may have faced possible stressors in real life, has also been encouraged by previous research [29]. Also, cognitive biases at different processing levels (attention, interpretation, and memory) have traditionally been studied independently from each other but some authors now state the need to know how they interact with each other [30, 31].

### Objectives and hypotheses

The purpose of the study is to design a brief online intervention aimed at reducing the interpretation of negative emotional cognitive biases. The intervention will be applied to an experimental group to analyse its impact on cognitive and emotional variables in comparison to a waiting list control group. More specifically, the intervention is framed within the field of CBM-I but, instead of using repetitive training, it is based on the techniques frequently used in cognitive-behavioural therapies (e.g. [31–33, 34] Leahy). Thus, while original CBM studies (e.g. [19]) were designed to train participants to change automatic processing of information with repetition of trials, the current intervention aims to teach participants the meaning and consequences of emotional cognitive biases, and how to modify them.

This study also aims to address some limitations of previous CBM procedures. First, based on previous evidence [35], mental imagery is used in order to shed some light on its role when modifying cognitive processes. Second, direct and indirect measures of cognitive performance are used to complement the information provided by self-report questionnaires. Moreover, these measures evaluate the three different domains proven to be most affected in emotional disorders (attention, interpretation, and memory) [36], given the need to explore their interplay [37]. Finally, a longitudinal follow-up was used to explore the continuation of benefits in time.

It is hypothesised that there will be a significant change in interpretation bias (less negative or more positive/neutral) from pre-intervention to post-intervention in the experimental group in comparison to the control group. It is also expected that changes in interpretation biases will also be associated with significant changes in attention and memory biases (less negative or more positive/neutral) from pre-intervention to post-intervention in the experimental group in comparison to the control group. Finally, it is hypothesised that the intervention will help the experimental group to reduce symptoms of depression and anxiety and increase well-being from pre-intervention to post-intervention in comparison to the control group. Due to the lack of conclusive findings from previous studies, it is explored whether these changes are maintained over time (after 2 weeks and 3 months) and the temporal dynamics of the different cognitive bias processes (attention, interpretation, and memory).

## Methods

### Participants and recruitment

Participants will be volunteer students from the Complutense University of Madrid who will be recruited via social media channels associated with the university and posters at the Faculty of Psychology. Inclusion criteria will be (1) aged 18 years or older and (2) being interested in the intervention, announced as a free online programme to ‘learn how to control the influence of thoughts on emotional reactions’. The announcement will include the name and email address of one of the researchers. Volunteers will be instructed to contact the researcher for more information, and academic course credits will be offered to those interested within the context of an official faculty programme that aims to promote the students’ involvement in academic training events. Exclusion criteria will include (1) having any form of visual and/or auditory disability that makes participants unable to follow online sessions and (2) lack of Internet access. No restrictions will be placed regarding concomitant treatment during the study, although this information will be monitored during the assessment sessions. When participants first contact the researcher, they will be explained all the details of the intervention and procedure. Inclusion and exclusion criteria will be evaluated by asking about their ability to correctly complete the programme (i.e. no disabilities and Internet access). The information about the intervention will also be given during their first visit to the laboratory in a printed document format before they are asked to sign the consent form.

### Randomisation and blinding

This study is a randomised superiority trial with two parallel groups and a 1:1 allocation ratio. Participants will be randomly allocated to the experimental group (CBM_Clin_) or a control waiting list group. This control group was chosen due to the novelty of the intervention, for which a waiting list is recommended to get a first impression of its effects [38]. Randomisation will be conducted by the main investigator using an Excel macro. This method assigns a different code to each group, which will only be known by the researchers. In this trial, solely participants will be blinded to their allocation and the meaning of codes.

### Sample size

The sample size was calculated based on the estimated effect size of the change in interpretation bias before and after a CBM-I intervention (*d* = .43) according to a recent meta-analysis on the field [20]. Following G*Power calculations [39], the minimum sample size (*α* set at 0.05, power at 0.95) to find a difference in a repeated-measures multivariate analysis of variance with one within-subjects factor (two-time points) and one between-subjects factor (two groups) was 73 participants. Due to expected attrition [40], 20% more participants will be recruited, reaching a total sample size of 88 participants.

### Intervention: ‘Relearning how to think’ programme

**Fig. 1:**
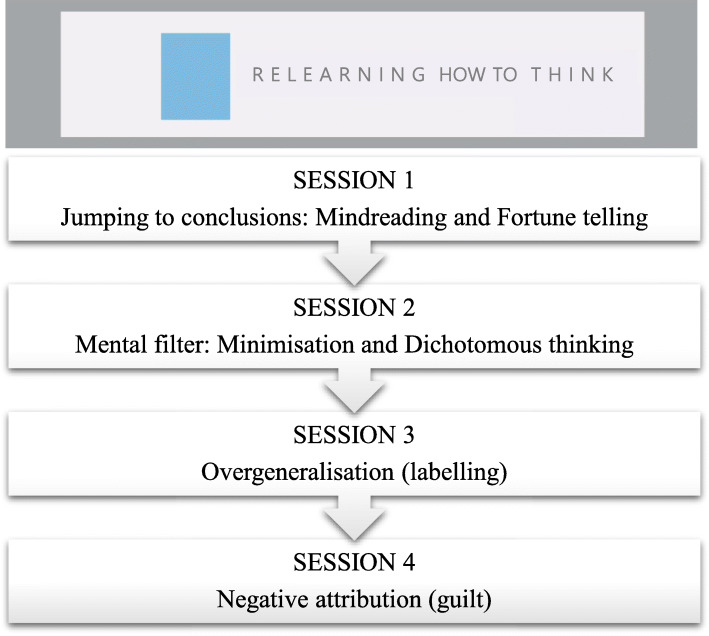
Classification of the specific cognitive biases targeted in the four online sessions of the programme

This is a brief online intervention designed to modify negative emotional interpretation biases. The intervention is composed of four different sessions in audiovisual format with psychoeducational content, open-answer questions, and exercises to be completed by users. Different cognitive biases, such as jumping to conclusions, mental filter, overgeneralisation, and negative attributions, are targeted in each session following classical descriptions of biases [7, 41]—see Fig. 1. The organisation of the content of the sessions was based on the Cognitive Error Rating Scales (CERS) [42], a manual created for therapists to evaluate cognitive errors during clinical sessions, and the CBM-errors [43], a clinical strategy to promote more benign interpretations following Beck’s theory [24].

**Fig. 2:**
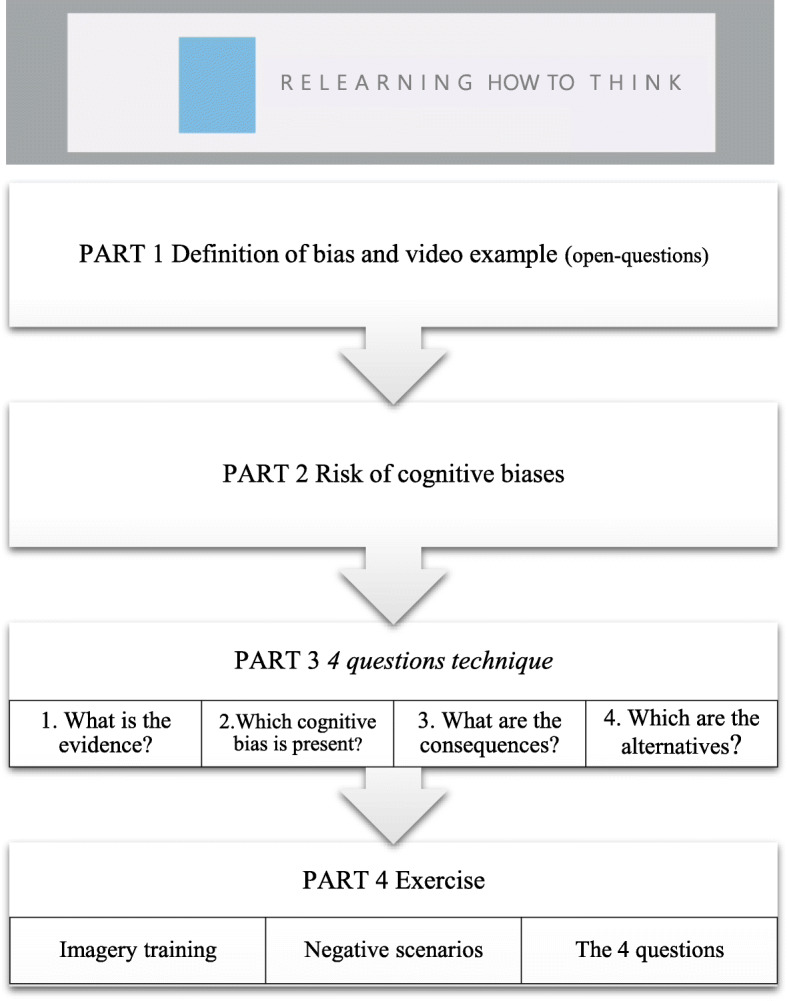
Structure of each session of the programme

**Fig. 3:**
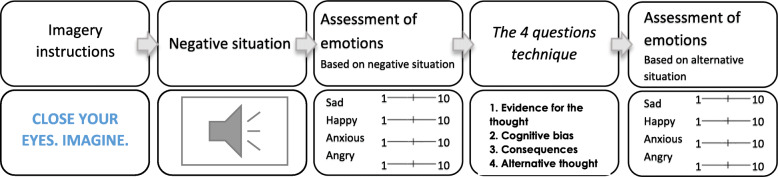
Practice exercise of each session of the programme

Each session of ‘Relearning how to think’ is composed of four different parts (see Fig. 2). In part 1, participants receive information about specific interpretation biases and are given examples in video format. Some of these videos are daily life scenes where professional actors represent examples of cognitive biases (following clinical vignettes described elsewhere) [6]. During each video (before the resolution of the scene), and to increase active involvement, participants have to complete an open-ended question about what could happen in those ambiguous situations. In the second part, users are informed about the risks of using negative interpretations, which is followed by an explanation of the strategies to avoid them in part 3. These strategies are based on ‘the 4-questions technique’ [44], widely used in clinical practice. This technique involves 4 steps to re-evaluate the negative interpretation of a given situation: 1) finding evidence for the negative interpretations, 2) uncovering the cognitive bias present in the situation, 3) identifying the negative consequences, and 4) creating alternative ways of thinking. Finally, during part 4, participants have the opportunity to practice the strategies in an exercise composed of imagery training [45] followed by negative scenarios in an audio format aimed to be reinterpreted. Figure 3 shows the steps of the exercise. It starts with an imaginary training [45] aimed to make scenarios more vivid to users. Participants are presented with a screen saying ‘Close your eyes. Imagine.’ for 1 s followed by a black screen during which a negative scenario is played in audio format with a female voice (e.g. *Your partner travels to work by car and normally arrives home promptly every day. Today you notice that they are over an hour late. Your first thought is that there must have been a crash*). Audio scenarios are daily life situations where negative interpretations arise, and participants are asked to imagine themselves in those situations. A beep is played for users to open their eyes and start with the exercise questions. First, they have to rate their mood (sadness, happiness, anxiety, and anger) on a 10-point VAS scale based on the most frequent emotions experienced in daily life [46, 47]. Then, they are guided to apply the 4 questions technique to each scenario. Finally, users evaluate the degree to which they believe in the new alternative thoughts and emotions generated by the new scenario (sadness, happiness, anxiety, and anger).

### Cognitive bias measures

#### Ambiguous Scenarios Test for Depression-II

The AST-D-II [48] is a self-report measure of interpretation biases. It consists of 15 ambiguous scenarios which participants have to rate on a scale from −5 (very unpleasant) to 5 (very pleasant). Participants are asked to imagine each scenario as vividly as possible and as if it was happening to them (e.g. *As you enter the room, the commission welcomes you and begins with the oral examination. After just a few minutes you know intuitively how the examination will go*). Two parallel versions are used in counterbalanced order at baseline, post-treatment, and follow-ups. Participants’ responses to the items were transformed into a total sum score by adding up the values of each item, rescaled from 1 to 11 (higher scores represent more positive interpretations). Internal consistency is good (*α* = 0.87) for the general scale and also for its two short versions A (*α* = 0.77) and B (*α* = 0.78).

#### Mouse-based (gaze) Contingent Attention Task (MCAT) [49]

A variant of the original Scrambled Sentence Test (SST) [50] is used to measure interpretation bias while monitoring attention towards emotional stimuli, based on the same principles as in the Eye-gaze Contingent Attention Training (ECAT) [51]. At the beginning of the task, participants have to click a fixation cross at the left side of the screen to elicit natural left-to-right reading patterns. Then, the *reading screen* is presented with a six-word emotional scrambled sentence (e.g. ‘am winner born loser a I’) where each word is hidden with a blank mask. Participants are instructed to move the mouse cursor over each mask to read the hidden word and mentally form a grammatically correct sentence using five of them. They are given a time limit of 14 s per sentence. This procedure is used to objectively measure attention biases toward emotional words (negative or positive). Then, the *answer screen* is presented with the six words unmasked for participants to click the order of the sentence they had mentally formed. In this section, participants are given a time limit of 7 s. Two are calculated from this procedure. First, the time spent (in ms) reading negative words divided by the total time spent (ms) reading positive and negative words is the index of overall negative attentional bias. Second, the resulting ratio of correctly unscrambled negative sentences and correctly unscrambled positive and negative sentences is considered to be the index of automatic negative interpretation bias [51]. In both cases, higher scores indicate higher negative cognitive biases. To maximise the appearance of biases, at the beginning of the task, participants are presented a six-digit number for 5 s and told to keep that number in mind during the entire task as they will be asked to retrieve it at the end of the task. This procedure will be completed by participants at both pre- and post-intervention evaluations.

#### Memory bias task

As a measure of memory biases, participants are given 5 min to remember the sentences they constructed during the MCAT procedure. Following Everaert et al.’s procedure [31], the ratio between negative sentences and the total number of emotional sentences recalled will be used as an index of negative memory bias. This procedure will be completed by participants at both pre- and post-intervention evaluations.

#### Computerised beads task [52]

The beads task is a measure of probabilistic reasoning which was initially designed to measure jumping to conclusion (JTC) bias in schizophrenic patients [53]. The adapted version used in this study has two parts. The first part consists of presenting two jars with beads of two different colours in different ratios (e.g. 60 orange/40 purple, and 60 purple/40 orange). Participants are told that the programme selects one of the jars to take beads randomly out of it and then return them. The instruction is to decide which jar is being used, based on the number of beads of each colour. The second part follows the same procedure with the difference being that the beads are all in white but present two different ratios of positive and negative adjectives (60 positive/40 negative, and 60 negative/40 positive). The number of beads viewed before reaching a decision is considered to be an index of jumping to conclusion bias. This procedure will be completed by participants at both pre- and post-intervention evaluations.

### Symptom measures

#### Depression, Anxiety and Stress Scale-21

The DASS [54] is a 21-item self-report questionnaire measuring symptoms of depression, anxiety, and stress. Each of the three subscales contains 7 items which, by adding up their values, provide a score for the three constructs. This questionnaire has shown good reliability with the following Cronbach’s alpha values for the Depression, Anxiety, and Stress scales, respectively: 0.84, 0.70, and 0.82 [55]. This procedure will be completed by participants at both pre- and post-intervention evaluations.

#### Patient Health Questionnaire-9

The PHQ-9 [56] is a 9-item self-report questionnaire to assess any present episodes of depression according to the DSM-IV diagnostic criteria. Each item is rated in frequency on a 4-point scale from 1 (not at all) to 4 (nearly every day). This questionnaire has shown good reliability with a Cronbach’s *α* of 0.89 [57]. An adapted PHQ-9 will also be used to measure past episodes of depression. The method of aggregation will consist in adding up the values of each item. In this study, the standard diagnostic cutoff score of PHQ-9 ≥ 10 [58] will be used to create groups based on present and past episodes of depression. This questionnaire will be completed online at pre-intervention to classify participants according to their symptom levels.

#### Generalised Anxiety Disorder-7

Generalised Anxiety Disorder-7 [59] is a 7-item self-report questionnaire to assess any present episodes of anxiety according to the DSM-IV diagnostic criteria. Each item is rated on a 4-point scale from 0 (not at all) and 3 (nearly every day), with the final score being between 0 and 21 (calculated by adding up the values of each item). An adapted version of this questionnaire was also used to measure past episodes of anxiety. The cutoff score used in this study to consider present or past episodes of anxiety was GAD-7 ≥ 10, following the severity scale: minimal (0–4), mild (5–9), moderate (10–14), and serious (14–20) [60]. This questionnaire will be completed online at pre-intervention to classify participants according to their symptom levels.

### Other measures

#### Pemberton Happiness Index

The PHI [61] is an 11-item self-report questionnaire measuring general, eudaimonic, hedonic, and social well-being. All the items will be summed up to reach a general well-being measure. It has been shown to have very good reliability (*α* = .92). This procedure will be completed by participants at both pre- and post-intervention evaluations.

#### Dysfunctional Attitudes Scale

The DAS [62] is a scale of 40 sentences reflecting dysfunctional cognitive schemas. Participants have to rate each sentence from 0 (not applicable to me) to 3 (highly applicable to me). The sum of the scores is considered to be an index of stable dysfunctional attitudes (i.e. a measure of cognitive structures)*.* Inverse items will be rescaled so that higher scores reflect higher levels of dysfunctional attitudes. The DAS is a predictor of major depression [63] and it has shown to have good reliability *α* = .70 [64]. This procedure will be completed by participants at both pre- and post-intervention evaluations.

#### Ruminative Responses Scale

The RRS [60] is composed of two subscales measuring rumination cognitive style. For the present study, only the 5-item brooding subscale will be used to measure the tendency to ruminate about negative events by adding up all the items. The scale has shown good reliability (*α* = .93) [65]. This procedure will be completed by participants at both pre- and post-intervention evaluations. *The scale for mood assessment-EVEA* [66] is a measure of current mood that participants take immediately before and immediately after each of the sessions of the programme. It is included to reflect some possible reactions to the cognitive training procedure. Participants have to rate, from 0 to 10, their current level of anger, happiness, anxiety, depression, and boredom. Scores of each subscale (4 items each) are summed up providing an index of emotional change during the session.

#### Credibility and Expectancy Questionnaire

The CEQ [67] is a 6-item measure used to assess the expectancy and rationale credibility of participants regarding the online programme they are offered before they start it. It consists of two subscales that measure credibility based on cognition (*what you think*) and treatment expectancy based on affect (*what you feel*). Both subscales have shown to have good internal consistency (*α* = .86 for credibility, *α* = .90 for expectancy).

#### The Working Alliance Inventory for Internet interventions (WAI-I)

The Working Alliance Inventory for Internet interventions (WAI-I) [68] is a self-report measure to assess alliance in Internet interventions. In this study, only the 8-item subscale of task and goal agreement with the programme was used to measure the level of concordance of the programme with participants’ interests. This measure was used at the end of the programme to know if participants were satisfied with the result. An example of an item is ‘Through the online program I have become clearer about the things I need to do to help improve my situation’. Cronbach’s *α* for this subscale has been found to be good (*α* = .84) [68].

#### The Stressful Events Questionnaire (SE)

The Stressful Events Questionnaire (SE) [69] is a self-report scale to measure stressful situations that happen to participants between the second assessment and the follow-up (2 weeks and 3 months). The scale includes positive and negative ratings of high-impact events as well as daily events related to different contexts (social, emotional, academic/occupational, and ‘other’).

### Procedure

**Fig. 4:**
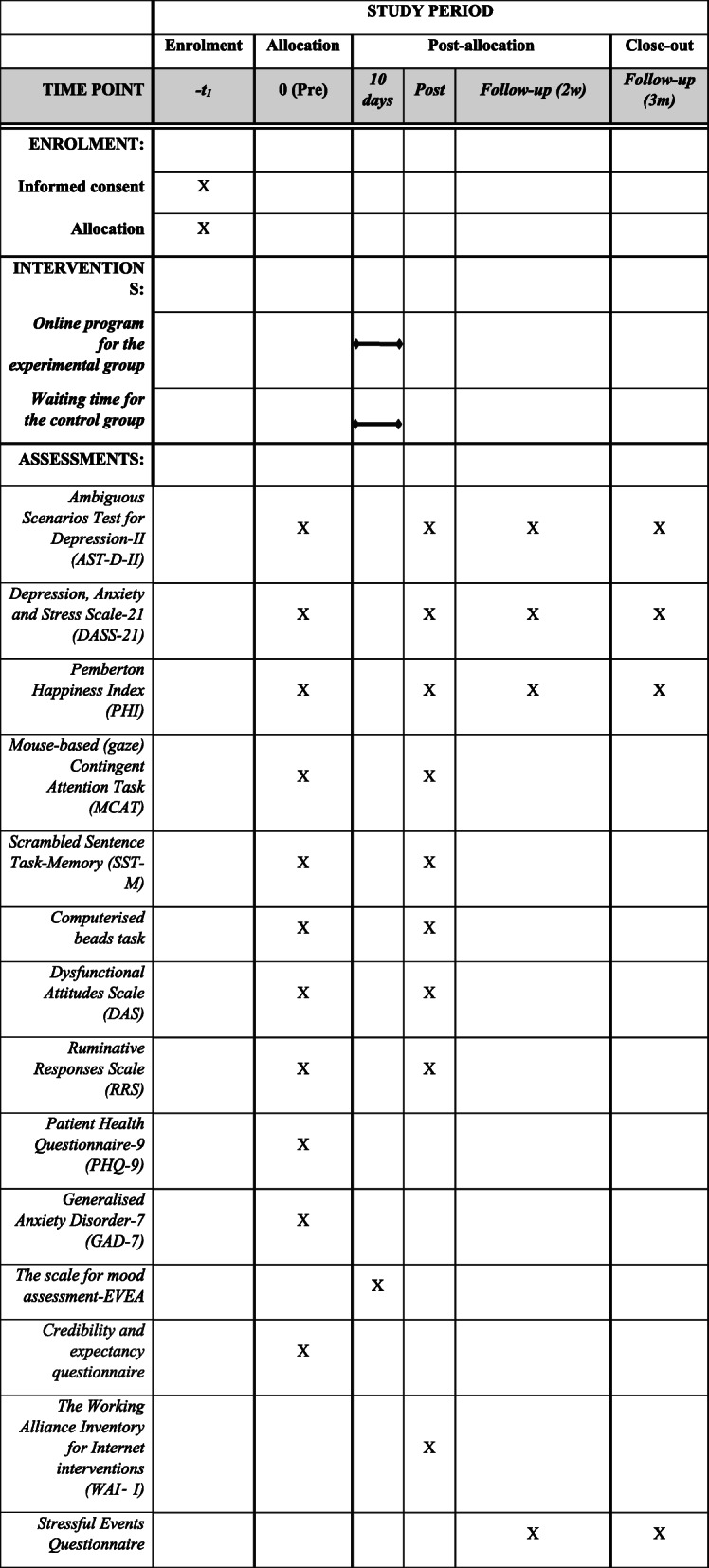
Schedule of enrolment, interventions, and assessments

Figure 4 shows the schedule of enrolment, intervention, and assessment following the recommendations for clinical trials [70]. The main investigator will create a random sequence to assign participants to either the experimental or the control group. After the volunteers contact the main researcher for initial information about the study, they will receive a phone call from the researcher to explain the procedure of the study and the outline of the intervention programme. Participants will be given all the ethical considerations regarding their participation and will be allowed to ask any further questions. After they verbally consent to continue, they will be assigned a participation code, randomly generated to ensure anonymity. Then, participants will complete two different assessments. First, they will receive the questionnaires for the first assessment (i.e. PHQ-9, GAD-7, AST-D-II, DASS-21, DAS, RRS, PHI, and CEQ), together with the information sheet providing details of the programme. Questionnaires will be completed online, using the Qualtrics platform, to which participants will be invited via a personalised link sent to their mailbox. Second, the day after participants complete these questionnaires at home, they will attend the first session in the lab. At that moment, they will be given the consent form (explaining their right to freely withdraw from the study at any moment) and will be asked to sign it if they agree to continue. Then, the main investigator will explain in detail the rationale of the intervention ‘Relearning how to think’ and will answer any questions from the participants. If they finally agree to participate, they will be asked to sign the consent form and complete some demographic information. Then, the three experimental tasks (MCAT, computerised beads task, and recall task) will be administered.

After completion of the three lab tasks, participants in the experimental group will receive in their email the link to complete the online programme from home. They will need to log in to an online platform created for this study and will be invited to create their own account and password to access the materials. Information will be stored solely using the participant’s code number and only the main researchers will have access to the data. These data will be recorded in the database of the platform and will be used by the researchers to monitor if participants in the experimental group complete all four online sessions before the post-intervention evaluation. When participants first access the platform, they will find only the first session available. Access to the next session will be granted only 24 h after completing the previous one. This intermission between sessions aims to enhance participants’ processing of the contents of each session as well as to avoid cognitive overload and boredom.

After finishing the 4-session training programme, participants in the experimental group will be notified again, after approximately 10 days, for a second assessment session. The control group will be allowed to complete the intervention right after the second assessment. To improve adherence, both groups will receive a reminder for the second assessment session the day before. Finally, participants will be sent the follow-up questionnaires (AST-D-II, DASS-21, PHI, SE) twice (in the next 2 weeks and 3 months). To increase adherence during the follow-ups, participants will be sent up to a maximum of three reminders to complete questionnaires. Both groups will have the opportunity to complete a ‘feedback question’ to give their opinion about the intervention. This question will be included in the post-assessment for the experimental group and at the follow-ups for the control group.

The procedure has been approved by the university ethics committee (Ref. 2018/19-017) and has been registered (ClinicalTrials.gov NCT03987477). Moreover, it follows the recommendations for a clinical trial protocol [70].

### Analytic plan

Demographic data and pre-treatment measures will be analysed to test for group differences with analysis of variance and chi-squared test for nominal variables.

Complete case analyses will be conducted for those participants who complete all 4 online sessions and attend pre- and post-assessment evaluation sessions. A series of 2 (group: experimental, control) × 3 (symptom group: never, present, past) × 2 (time: pre-training, post-training) analyses of variance will be performed to evaluate the change between groups. The symptom group will be created based on present and past symptoms of depression and/or anxiety to explore their influence on the results. Intention-to-treat (ITT) analyses will be conducted with all participants, regardless of session or outcome measure completion. ITT mixed models (restricted maximum likelihood (REML) estimation) will be used to account for missing data [71]. Binary logistic regression will be used to evaluate the assumption that data is Missing at Random (MAR). Exploratory mediation analyses will be conducted to study the interplay between the different cognitive bias scores given the change of the intervention. Finally, follow-up assessments will be included in a series of analyses of variance to evaluate group differences in time. All analyses will be performed in SPSS Statistics 20 with an *α* level of 0.05.

## Discussion

The current study will test the efficacy of a brief online intervention to target emotional negative cognitive biases. Although traditional CBM interventions are designed to change this type of dysfunctional processing in an automatic way [17], the rationale of ‘Relearning how to think’ is to increase participants’ awareness of their interpretation biases and guide them to change these biases , in a more effortful way, by following a clinically oriented working frame (CBM-I_Clin_).

The study uses a transdiagnostic conceptualisation of the role of cognitive biases in psychopathology. Given the high comorbidity between anxiety and mood disorders [1], having intervention tools that can tap both problems could be clinically useful. Some of the video scenarios designed for the programme were based on examples proposed by previous transdiagnostic approaches [6], and the content is suitable for different common psychopathologies in which cognitive biases may play a role.

The study also addresses some of the questions that remain unanswered regarding CBM efficacy. It combines subjective (e.g., AST-D) along with objective measures (e.g., MCAT) to assess cognitive biases. The present study aimed to complement both self-report and behavioural measures to avoid potential biases of respondents. Mental imagery is also used following the proposal that it may enhance CBM-I performance [14]. Holmes et al. [45] found that mental imagery of emotional content has a beneficial impact on cognitive change. ‘Relearning how to think’ includes imagery training to potentiate vividness of the scenarios and promote interpretation change.

Furthermore, it explores innovatively whether changes in interpretation biases may be associated with concomitant changes in attention and memory. There is very scarce basic research on the interrelation between different types of biases in emotional disorders [31], and this study will offer a unique opportunity to explore whether a specific intervention designed to change the interpretation of ambiguous scenarios may also affect other domains of information processing.

Finally, there is an increasing interest in the use of online interventions and many researchers wonder if this format is also beneficial for individuals with clinical problems. The extant evidence suggests that psychological treatments delivered online can be as effective as face-to-face therapies [72, 73] and seem to overcome some of the limitations traditional therapies present [74]. For example, online sessions can be taken by the individual at any time, there is no need to wait to schedule dates, stigma is reduced, and individuals increase their self-efficacy [75]. Specifically, CBM interventions seem to be highly suitable for the online format due to its flexibility in application, or the minimal requirement of supervision, in comparison to traditional therapies. CBM could even be used in a self-management way so that it could be applied to patients waiting for treatment or presenting vulnerability factors [28]. In sum, we expect that this study will offer new responses to some of the challenges CBM procedures face to make them more feasible, efficient, and more capable of providing answers to some theoretical issues related to the complex relations between emotion, cognition, and clinical psychology.

## Trial status

The trial was registered on June 17, 2019, with ClinicalTrials.gov NCT03987477. The study started recruiting participants on September 30, 2019, and was completed by December 30, 2020.

https://clinicaltrials.gov/ct2/show/NCT03987477?term=vazquez+and+nieto&draw=2&rank=1

## Data Availability

Not applicable.

## Abbreviations

ABM: Attentional Bias Modification
AST-D-II: Ambiguous Scenarios Test for Depression-II
CBM: Cognitive Bias Modification
CBM-I: Cognitive Bias Modification of Interpretations
CBM-I_Clin_: Cognitive Bias Modification of Interpretations based on clinical procedures
CEQ: Credibility and Expectancy Questionnaire
DAS: Dysfunctional Attitudes Scale
DASS-21: Depression, Anxiety and Stress Scale-21
ECAT: Eye-gaze Contingent Attention Training
EVEA: Scale for mood
GAD-7: Generalised Anxiety Disorder Scale-7
ITT: Intention-to-treat
JTC: Jumping to conclusions
MANOVA: Multivariate analysis of variance
MAR: Missing At Random
MCAT: Mouse-based (gaze) Contingent Attention Task
PHI: Pemberton Happiness Index
PHQ-9: Patient Health Questionnaire-9
REML: Restricted Maximum Likelihood
RRS: Ruminative Responses Scale
SE: Stressful Events Questionnaire
SST: Scrambled Sentence Test
WAI-I: The Working Alliance Inventory for Internet interventions
